# A team approach to improving colorectal cancer services using administrative health data

**DOI:** 10.1186/1478-4505-10-4

**Published:** 2012-01-31

**Authors:** Geoffrey Porter, Robin Urquhart, Jingyu Bu, Cynthia Kendell, Maureen MacIntyre, Ron Dewar, George Kephart, Yukiko Asada, Eva Grunfeld

**Affiliations:** 1 Queen Elizabeth II Health Sciences Centre, Halifax, Nova Scotia, Canada; 2 Department of Surgery, Dalhousie University, Halifax, Nova Scotia, Canada; 3 Department of Community Health and Epidemiology, Dalhousie University, Halifax, Nova Scotia, Canada; 4 Cancer Outcomes Research Program, Cancer Care Nova Scotia, Halifax, Nova Scotia, Canada; 5 Surveillance and Epidemiology Unit, Cancer Care Nova Scotia, Halifax, Nova Scotia, Canada; 6 Ontario Institute for Cancer Research, Toronto, Ontario, Canada; 7 Department of Family and Community Medicine, University of Toronto, Toronto, Ontario, Canada

**Keywords:** Colorectal cancer, interdisciplinary research, health services, administrative health data

## Abstract

**Background:**

Colorectal cancer (CRC) is the third most commonly diagnosed cancer in Canada and accounts for 11.9% of all cancer-related mortality. Fortunately, previous studies have provided evidence of improved outcomes from access to timely and appropriate health services along the disease trajectory in CRC. As a result, the CIHR/CCNS Team in Access to Colorectal Cancer Services in Nova Scotia (Team *ACCESS*) was created to build colorectal cancer (CRC) research capacity in Nova Scotia (NS) and to study access to and quality of CRC services along the entire continuum of cancer care.

**Objectives:**

The objectives of this paper are to: 1) provide a detailed description of the methodologies employed across the various studies being conducted by Team *ACCESS*; 2) demonstrate how administrative health data can be used to evaluate access and quality in CRC services; and 3) provide an example of an interdisciplinary team approach to addressing health service delivery issues.

**Methods:**

All patients diagnosed with CRC in NS between 2001 and 2005 were identified through the Nova Scotia Cancer Registry (NSCR) and staged using the Collaborative Stage Data Collection System. Using administrative databases that were linked at the patient level, Team *ACCESS *created a retrospective longitudinal cohort with comprehensive demographic, clinical, and healthcare utilization data. These data were used to examine access to and quality of CRC services in NS, as well as factors affecting access to and quality of care, at various transition points along the continuum of care. Team *ACCESS *has also implemented integrated knowledge translation strategies targeting policy- and decision- makers.

**Discussion:**

The development of Team *ACCESS *represents a unique approach to CRC research. We anticipate that the skills, tools, and knowledge generated from our work will also advance the study of other cancer disease sites in NS. Given the increasing prevalence of cancer, and with national and provincial funding agencies promoting collaborative research through increased funding for research team development, the work carried out by Team *ACCESS *is important in the Canadian context and exemplifies how a team approach is essential to comprehensively addressing issues surrounding not only cancer, but other chronic diseases in Canada.

## 1. Background

### 1.1. Colorectal Cancer

In Canada, an estimated 22,200 new cases of colorectal cancer (CRC) were diagnosed in 2011, making it the third most commonly diagnosed cancer in both men and women in Canada [1]. Despite a modest decrease in national mortality rates over the last 20 years, CRC mortality still accounts for approximately 11.9% of cancer-related deaths [1].

Nova Scotia (NS) has the second highest incidence rate of CRC of all Canadian provinces, with an estimated 830 new cases in 2011 [1]. Interestingly, estimated mortality rates are higher (30 vs. 25 per 100, 000 people) [1] and estimated 5-year relative survival rates are lower in NS (56% vs. 62%) [2] compared to Canada, suggesting poorer patient outcomes for CRC patients in NS than other provinces. Previous studies have provided evidence of improved outcomes from access to timely and appropriate health services along the disease trajectory in CRC and other malignancies [3,4], highlighting the need for improved research into CRC services in NS.

### 1.2. Team *ACCESS*

Team *ACCESS *was formed in 2007 after receiving a New Emerging Team (NET) grant from the Canadian Institutes of Health Research (CIHR). These grants are awarded to encourage the formation of new research teams and to build capacity in new and developing areas of research. Team *ACCESS *is an interdisciplinary team consisting of more than 20 researchers and decision-makers (i.e., clinicians, program managers/directors, policy-makers) from a broad range of methodological and health disciplines. Collectively, Team *ACCESS *possesses expertise in health services research, epidemiology, biostatistics, population health, health administration, primary care, psychiatry, pediatrics, pathology, and surgical, medical, and radiation oncology. Through this unprecedented collaborative research effort in NS, Team *ACCESS *has sought to improve the capacity to examine access to and quality of CRC services at transition points across the entire continuum of cancer care (i.e., presentation of signs/symptoms, diagnosis, surgery, systemic and radiation therapy, follow-up care, and advanced disease/palliative care). Such transitions may be particularly susceptible to problems of access and quality since they often require patients to move across health care sectors as part of a complex and fragmented delivery system [5].

The specific research objectives of Team *ACCESS *are to; 1) develop tools to measure and improve timely and equitable access to, and quality of, CRC services along the cancer care continuum, 2) explore methods to integrate access and quality relevant to CRC services, and measure the impact on outcomes, and, 3) develop and test methods for the knowledge transfer of findings towards improving access to quality CRC care.

Importantly, at the time this award was received, Team *ACCESS *was the only team in Canada that was examining CRC health services across the entire continuum of care. After four years of research in this area, we know of no other group in Canada that is examining CRC in such a comprehensive manner.

## 2. Objectives

The aim of this paper is to provide an account of the expertise and preliminary work required to study CRC services along the cancer care continuum. Specifically, the objectives of this paper are to: 1) provide a detailed description of the methodologies employed across the various studies being conducted by Team *ACCESS*; 2) demonstrate how administrative health data can be used to evaluate access and quality in CRC services; and 3) provide an example of an interdisciplinary team approach to addressing health service delivery issues.

## 3. Methods

Team *ACCESS *has embarked on more than 20 studies to date (Table 1), with most now completed or nearing completion. These studies span the entire continuum of care, from presentation of signs/symptoms, to diagnosis, treatment, follow-up care, and advanced disease/palliative care. While the methods used may vary from study to study (depending on the research question), the methods presented in this section are common to all studies.

**Table 1:** Summary of Team *ACCESS *research

Area of Study	Research Question/Study	Theme(s)
From screening/symptoms to diagnosis	What are the peri-diagnostic time intervals for CRC care? What factors affect receipt of timely care?	Access
	What factors related to pre-CRC diagnosis health care utilization are associated with late stage diagnosis of CRC?	Access, Quality
	Using micro-simulation, how will introduction of a population-based screening program impact on resource and health services utilization?	Access

From diagnosis to surgery	What are the surgical time intervals for CRC care? What factors affect receipt of timely care?	Access
	Are there differences in the use of health care services for emergency vs. elective presentation of CRC?	Access, Quality
	Has adequate lymph node assessment improved over time?	Quality
	What are the effects of lymph node assessment on overall CRC survival?	Quality
	Exploring Stage IIB survival: can we identify factors associated with poor outcomes in Stage IIB patients?	Access, Quality
	Timely access to and quality of care in CRC: are they related?	Access, Quality

From surgery to adjuvant treatment (chemotherapy/ radiotherapy)	What are the treatment time intervals for adjuvant CRC care? What factors affect receipt of timely care?	Access
	Is adjuvant therapy administered in accordance with established clinical practice guidelines?	Access, Quality
	What are the reasons for non-adherence to guidelines?	Quality
	Does inequity exist in meeting established benchmarks for radiotherapy and in receipt of treatment?	Access, Quality
	What is the completeness of colonoscopy, surgery, and pathology reporting for rectal cancer patient receiving radiotherapy?	Quality

From adjuvant treatment to follow-up care	What are the patterns of follow-up care for CRC?	Access, Quality
	Is follow-up care being provided according to established clinical practice guidelines?	Access, Quality
	How do patients feel about the follow-up care they receive?	Access, Quality
	Are CRC survivors receiving appropriate screening services for other cancers?	Access

End-of-life care	What are the patterns of medication use amongst end-of-life CRC patients?	Access, Quality
	Does inequity exist in health care utilization for CRC patients at end-of life?	Access

Knowledge Translation	Which approaches/strategies are useful in integrating research findings into clinical practice?	

### 3.1. Cohort Identification

**Figure 1:**
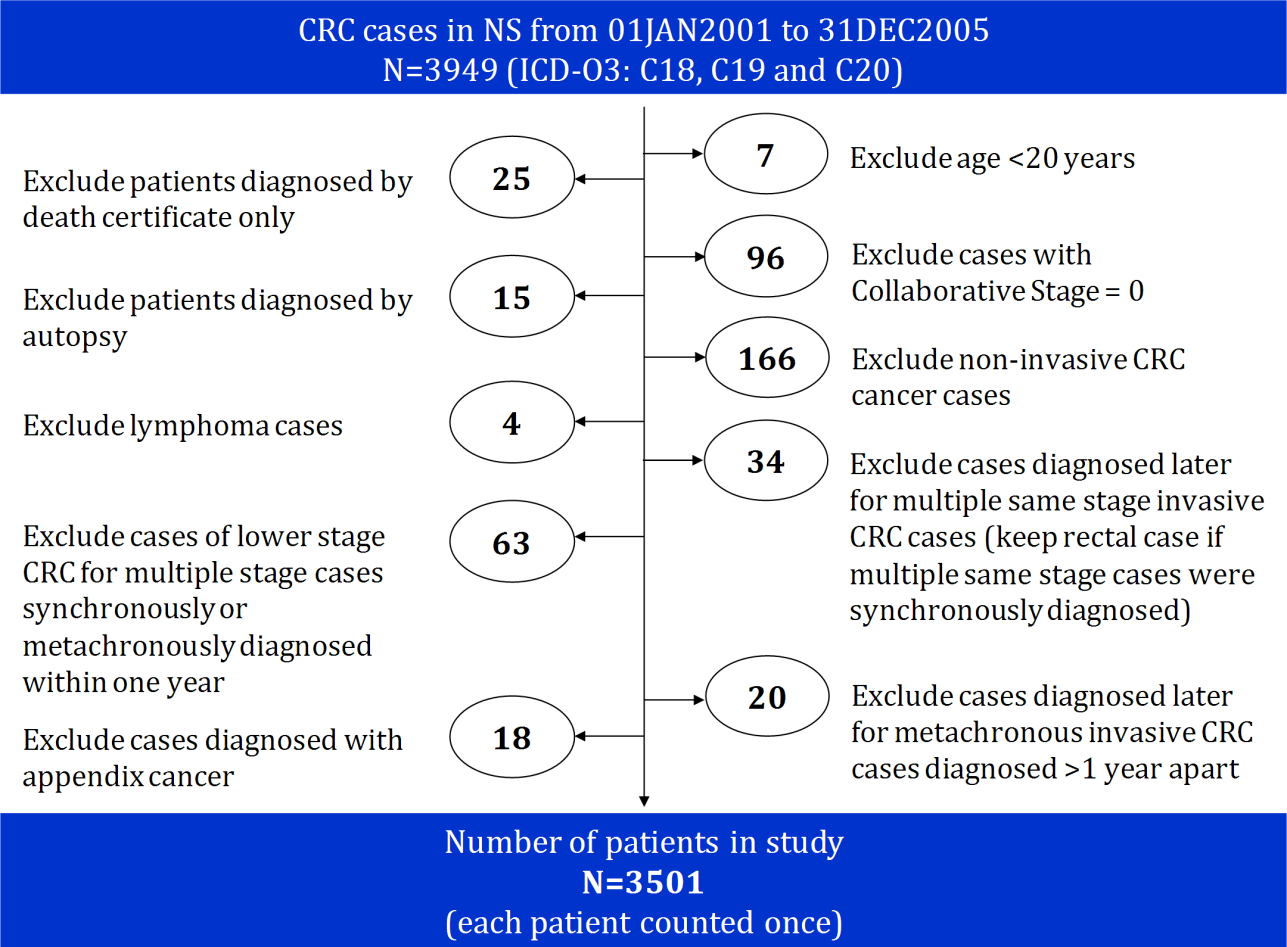
**Cohort identification**.

To maximize generalizability, a population-based cohort was identified. All incident cases of CRC (ICD-O-3: C18, C19, C20) in NS between 2001 and 2005 were identified through the Nova Scotia Cancer Registry (NSCR). To define the study cohort, we excluded; 1) cases diagnosed by death certificate only or autopsy, 2) individuals less than 20 years of age, and 3) diagnoses of appendix cancer (ICD-O-3: C18.1), lymphoma, non-invasive CRC, and invasive Stage 0 CRC [6]. For patients with more than one CRC diagnosis in the time period, we kept only one record per patient (Figure 1). Of the selected 3,501 patients that comprise the final study cohort, 2,385 (68.1%) had colon cancer and 1,116 (31.9%) had rectal cancer. Their demographic and disease characteristics are described in Table 2.

**Table 2:** Characteristics of study cohort

Cohort Characteristics	n (%)
Sex	
Female	1634(46.7)
Male	1867(53.3)

Age at Diagnosis	
20-64	1122(32.0)
65-74	941(26.9)
75+	1438(41.1)

Rural/urban residence	
Rural	1424(40.7)
Urban	2077(59.3)

Comorbidity score	
0	2031(58.0)
1+	1470(42.0)

Collaborative stage	
I	659(18.8)
II	1069(30.5)
IIA	882(25.2)
IIB	187(5.3)
III	916(26.2)
IIIA	88(2.5)
IIIB	555(15.9)
IIIC	273(7.8)
IV	697(19.9)
Unknown	160(4.6)

### 3.2. Cohort Staging

Since treatment for CRC depends on stage at diagnosis [7], stage data are key in examining issues surrounding timely receipt of quality care and the impact on patient outcomes. In Canada, staging data have not historically been collected by provincial cancer registries [8], as was the case in NS. Upon cohort identification, Team *ACCESS *collaborated with experienced coders at the NSCR to conduct an extensive chart review and stage all CRC cases using the Collaborative Stage (CS) Data Collection System, resulting in CS derived AJCC stage groups [6,9]. CS uses information from multiple sources to determine a "best" stage, effectively reducing the number of unknown stages within a population [10], contributing to the completeness of our data.

### 3.3. Data Sources

At the center of Team *ACCESS *research is a longitudinal 'database' assembled by linking individual patient records across the administrative health databases used in the study. Complete data (i.e. data from all databases) are available from January 1, 1999 to March 31, 2008. The earlier years of data permit the study of healthcare utilization for the two years preceding diagnosis for all patients in the cohort. Table 3 presents the databases used by Team *ACCESS*, as well as the main variables extracted from each database.

**Table 3:** List of administrative health databases accessed and the main variables extracted

Database	Variables
Nova Scotia Cancer Registry (NSCR)/Oncology Patient Information System	■ Patient demographics ■ Diagnosis and staging ■ Cancer center referrals and visits ■ Treatment information ■ Previous and subsequent primary cancers ■ Cancer recurrence
Mental Health Outpatient Information System (MHOIS)	■ Mental health clinic visits ■ Demographics ■ Diagnoses

Medical Service Insurance Physician Services (MSIPS)	■ Physician visits ■ Diagnoses ■ Procedures

Medical Service Insurance (MSI) Insured Patient Registry	■ Patient demographics ■ Enrollment status ■ Program eligibility dates

Licensed Provider Registry	■ Physician demographics ■ Physician specialty ■ Educational information

Vital Statistics	■ Patient demographics ■ Dates of death ■ Cause of death

Discharge Abstract Database (DAD)	■ Hospital discharges ■ Diagnoses ■ Procedures

Canadian 2001 Census Data	Neighborhood or community level measures: ■ Education ■ Income ■ Rural/urban residency

Seniors' PharmaCare Prescriptions (SP)	Prescription data for individuals 65 years and older: ■ Drug ID ■ Cost ■ Amount

Community Services Prescriptions (CSP)	Prescription data for individuals less than 65 years who are enrolled in a provincial income assistance program: ■ Drug ID ■ Cost ■ Amount

Nova Scotia Breast Screening Program	■ Procedure dates ■ Procedure types ■ Screening results ■ Diagnoses

Cervical Cancer Prevention Program	■ Smear dates ■ Diagnosis dates ■ Healthcare professional specialty

Capital Health Radiology Department (CHRD)	■ Radiology procedure codes ■ Procedure dates

Palliative Care Programs (PCP)*	■ Referral/enrollment date into a formal PCP

* Only available for the two districts with formal PCPs (i.e., Capital District Health Authority and Cape Breton District Health Authority)

As previously discussed, the cohort was identified through the NSCR. In NS, all new cancer diagnoses are reportable to the NSCR by law. The NSCR is operated by the Surveillance and Epidemiology Unit (SEU) at *Cancer Care Nova Scotia *(CCNS) and has data dating back to 1964. In NS, the SEU houses both the NSCR and the Oncology Patient Information System (OPIS), the administrative scheduling system for the province's two tertiary care cancer centres, resulting in a single database that contains both cancer registry and cancer centre data. Thus, for each patient included in the cohort, the following information was extracted from NSCR/OPIS: patient demographics, previous and subsequent primary cancers, diagnosis and staging, cancer center referrals, treatment-related and cancer recurrence visits, and radiotherapy treatments.

Through the Population Health Research Unit (PHRU) at Dalhousie University, we accessed and linked information from a variety of administrative health care databases [11]. The NS Department of Health (DoH) supplies PHRU with complete Medicare and hospital files suitable for research purposes. In accordance with DoH policy, all data held at PHRU uses an encrypted health card number to maintain patient confidentiality.

Other databases, held at individual departments/programs, were used by Team *ACCESS *to obtain additional information relevant to CRC services and/or patients. The databases accessed include; Capital District Health Authority's Radiology Department (CHRD) database, Capital District Health Authority's and Cape Breton District Health Authority's Palliative Care Program (PCP) databases, the CCNS Cervical Cancer Prevention Program (CCPP) database, and the Nova Scotia Breast Screening Program (NSBSP) database.

### 3.4. Data Linkage

**Figure 2:**
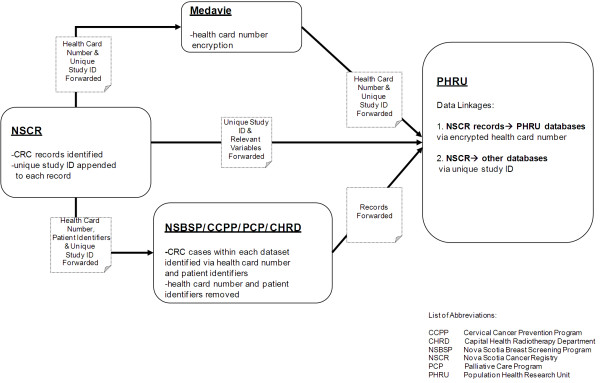
**Data linkage**.

To facilitate data linkage, a unique study ID was appended to each of the identified NSCR records. As shown in Figure 2, data linkage consisted of two processes:

1) To link NSCR data to PHRU data, the health card number (HCN) and unique study ID from the identified records were sent to the provincial healthcare administrator for HCN encryption. The unique study ID and encrypted HCN were then sent to PHRU. The information contained within the NSCR records (i.e., staging, demographics, cancer clinic visit activity, etc.) was sent directly to PHRU with only the unique study ID as a person-level identifier. Using the unique study ID, the encrypted HCN was re-assigned to the corresponding patient record to enable linkage to PHRU data.

2) To link NSCR data to the remaining databases, patient identifiers (i.e., HCN, date of birth, surname) and unique study ID for all CRC patients were sent to the dataset custodians at CHRD, PCP, CCPP, and NSBSP. All records that corresponded to patients in our cohort were identified within each database using the HCN and confirmed using the patient identifiers. The data custodian then extracted these records, removed all identifying information from the file, except the unique study ID, and sent the file to PHRU for linkage to NSCR records via unique study ID.

### 3.5. Measures

#### 3.5.1. Overarching Themes

The themes of access and/or quality are explored in each Team *ACCESS *study (see Table 1), with the overall objectives of integrating access and quality relevant to CRC services, and developing tools to assess these dimensions of the healthcare system.

Access to care is measured in terms of equity [12] and timeliness [13]. Specifically, Team *ACCESS *uses a needs-based approach [14] to determine whether those who should be receiving care, according to clinical practice guidelines (CPGs) and patients' needs profiles, are receiving it in a timely manner (according to CPGs and established benchmarks). At each stage along the cancer care continuum, the effect of various factors (i.e., age, sex, clinical, geographical and socioeconomic factors, and special needs of vulnerable populations [15]) on access to CRC services is investigated. Quality is assessed using documented cancer care quality indicators (QIs) [16] and/or adherence to CPGs. Where CPG adherence is low, reasons for non-adherence are being explored. Such reasons have been studied using the linked administrative data or, in certain situations, have required a focused chart review. To maintain patient confidentiality, chart reviews are undertaken in partnership with the NSCR, which holds the key for the unique study ID, with data subsequently linked in the manner described above.

#### 3.5.2. Covariates

All of our studies examine the influence of geography (e.g., urban/rural residence, health district, and distance to specialized services) as well as other patient characteristics (e.g., age, sex, cormorbidity, and neighborhood socioeconomic status) on the access and quality measure studied. To maintain methodological consistency across studies, team members collaborated to define the following covariates:

1) *Comorbidity *is assessed using a comorbidity score and a cancer history variable. A modified list of comorbidities was created using those identified by Elixhauser et al [17], with coding (ICD-9-CM and ICD-10) developed by Quan et al [18]. Cancer-related comorbidities were excluded, resulting in a list of 28 comorbidities. Using this list, an unweighted comorbidity count was obtained for each individual by examining hospital discharge records for two years prior to the index CRC diagnosis to 30 days after diagnosis (potential score: 0-28). Using NSCR data, a cancer history variable was created by counting each individual's previously diagnosed primary cancers. The comorbidity score and the cancer history variable are used as separate covariates for statistical analyses.

2) In addition to examining several variables independently (e.g., median household income, education), we use a deprivation index to measure *socioeconomic status*. This index includes both material deprivation and social deprivation as previously described by Townsend [19]. Using the "Quebec Model" [20], six indicators of material and social deprivation (proportion of persons with a high school diploma, employment/population ratio, average income, proportion of persons living alone, proportion of single parent families, and proportion of separated, divorced, and widowed persons) were extracted from census data. These indicators were used to generate material and social deprivation values for patients' census dissemination areas (DAs). Index values range between 0 and 4, with 4 being the least deprived. Patient postal codes at the time of diagnoses are linked, via the Postal Code Conversion File Plus (PCCF^+^) [21], to a specific DA (as defined by 2001 census data) and assigned the associated deprivation index values for that area.

3) *Rurality *is defined using the Statistical Area Classification (SACtype) and the Metropolitan and Census Agglomeration Influenced Zones (MIZ) classification developed by Statistics Canada to distinguish rural and urban areas [22,23]. Patients' postal codes obtained at the time of diagnoses were inputted into the PCCF^+ ^[21] to obtain a SAC type/MIZ value. If a patient resides in a census metropolitan area (CMA) or census agglomeration (CA), or in a zone that is strongly influenced by a CMA or CA, his/her location of residence is considered urban. A rural residence is defined as being located in a zone that is not strongly influenced by a CMA or CA.

## 4. Results/Discussion

The development of Team *ACCESS *represents a unique approach to cancer research, and specifically to CRC research. Prior to Team *ACCESS*, a formal research collaboration of this magnitude had not existed in NS, limiting the quality and breadth of CRC research conducted in the province. In addition, collaborations and partnerships have been established with local, provincial, and national cancer groups/organizations that further improve our research capacity by providing opportunities for dialogue on CRC and ongoing feedback, and thus contributing to our understanding of the issues that affect the cancer care system and the patients who use it.

Overall, Team *ACCESS *has made substantial contributions to CRC research. The first notable contribution being the development of research capacity in NS by assembling an interdisciplinary team, mentoring clinicians (with limited research experience), and training graduate students (MSc, PhD, and post-doctoral fellows) in cancer health services research. Second, by staging all CRC cases diagnosed in NS between 2001 and 2005, population-level stage data are available for a 5-year time period. Since treatment for CRC depends upon the stage at diagnosis [7], such data are vital to examining quality of care issues in CRC. Third, the creation of the Team *ACCESS *database linkage permits a thorough assessment of health system utilization in relation to CRC across the entire continuum of cancer care.

In addition to conducting research, Team *ACCESS *is focused on effecting change in the health care system through knowledge translation (KT) activities and collaborations with leaders in cancer research and cancer services. Specifically, we have taken an integrated KT approach by involving decision-makers in the development, conduct, and interpretation phases of research. We anticipate this approach will advance CRC care in the province in two ways: 1) by helping decision-makers facilitate changes in CRC programs and services based on the research findings; and 2) by helping researchers align their research questions and studies with issues relevant to the local decision-making communities.

These contributions illustrate how a team approach is essential to improving colorectal cancer services in NS. Our assessment of inequity in the colorectal cancer care system further exemplifies the importance of a team approach. Adopting a needs-based approach to inequity analysis, and moving beyond studying variations in care to understanding whether the variations are based on patients' *needs *or some non-need factors (e.g., sex, socio-economic status), has required expertise from a diverse team - specifically, researchers and analysts to facilitate database linkage, experts in inequity analysis and the specific analytic techniques (e.g., horizontal inequity index), oncologists with specific clinical knowledge (e.g., related to curative and palliative therapies) to help define need versus non-need variables and then appropriately interpret the findings, and individuals with knowledge translation experience to help translate the findings and their implications to the clinical and decision-making communities.

Our study of inequity moved beyond studying variations in care, to examining whether these variations in care are based on patients' *needs *or some non-need factors (e.g., sex, socio-economic status). This needs-based approach to inequity analysis has required expertise from a diverse team- researchers and analysts to facilitate database linkage, experts in inequity analysis and the specific analytic techniques (e.g., horizontal inequity index), oncologists with specific clinical knowledge (e.g., related to curative and palliative therapies) to help define need versus non-need variables and then appropriately interpret the findings, and individuals with knowledge translation experience to help translate the findings and their implications to the clinical and decision-making communities.

Our research has identified several limitations in data availability and capture in our province. Specifically, direct access to laboratory data was not available and chemotherapy data were incomplete. Regarding the latter, chemotherapy can be administered on an outpatient basis, often by nurses [8,24], without an associated physician billing. We have performed a chart review to obtain complete chemotherapy data. Moreover, several of the databases are not population-based (i.e., CHRD, PCP, and PharmaCare), but rather are limited to certain jurisdictions or patient populations (e.g., those 65 years of age and older), limiting our capacity to report upon diagnostic imaging, palliative care, and medication use for the entire province.

Team *ACCESS *developed out of a recognized need to study and improve access to quality CRC services in NS. The significance of this team is underscored by the involvement of many key decision/policy-makers, clinical leaders, and senior researchers from other disciplines (e.g., epidemiology, database analysis, equity analysis) to work on this topic. We anticipate that the skills, tools, and knowledge generated from our work will also advance the study of other cancer disease sites in NS. Our approach may also be adapted to study other chronic conditions and understand issues regarding access to and quality of care, and their impact on outcomes, in these patient populations.

## Competing interests

The authors declare that they have no competing interests.

## Authors' contributions

GP and RU were involved in the conception of Team *ACCESS*, conception and design of specific research studies, and analysis and interpretation of data. JB was involved in the design of several individual studies, analysis and interpretation of data, and manuscript preparation. CK was involved the coordination of studies, interpretation of data, and drafting of the manuscript. MM was involved in cohort identification, database linkage, and analysis and interpretation of data. RD was involved in cohort identification, database linkage, the development of common methodologic structures and tools and analysis and interpretation of data. GK was involved in the conception of Team access, development of common metholodgic structures and tools. YA was involved in development of common metholodgic structures and tools. EG was involved in the conception of Team *ACCESS *and conception and design of specific research studies. All authors revised the manuscript critically for important intellectual content and approved the final manuscript.
